# Supplementary figures and images for: Mental health and burnout during medical school: Longitudinal evolution and covariates
Source: PLoS One. 2024 Apr 16;19(4):e0295100. doi: 10.1371/journal.pone.0295100 (PMC11020803; doi:10.1371/journal.pone.0295100)

**S1 Fig. Participation flow chart**

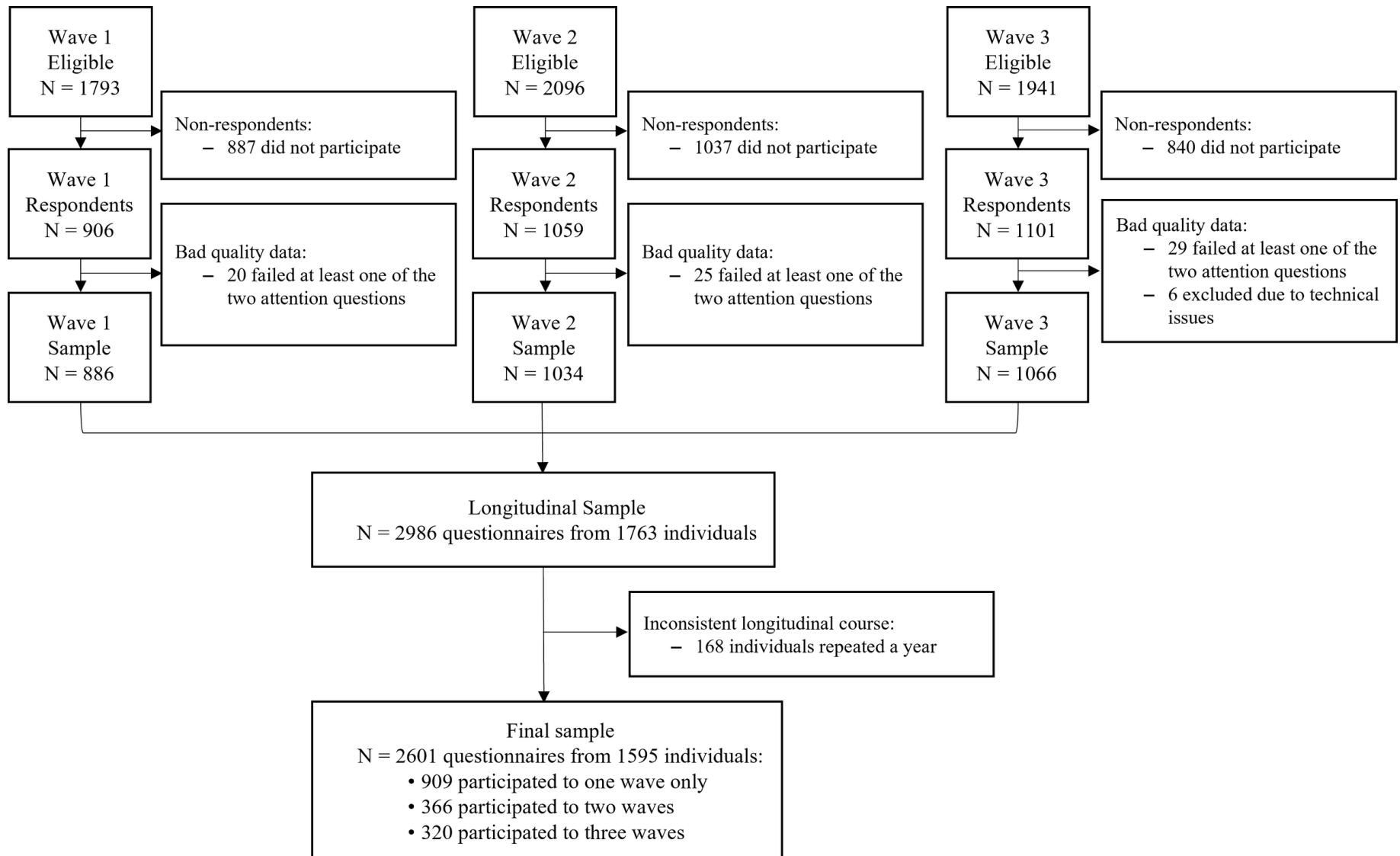

Supplement: S1 Fig — (PDF) [file pone.0295100.s001.pdf]
