# Supplementary material for: Mental health and burnout during medical school: Longitudinal evolution and covariates
Source: PLoS One. 2024 Apr 16;19(4):e0295100. doi: 10.1371/journal.pone.0295100 (PMC11020803; doi:10.1371/journal.pone.0295100)
Supplement: S1 Table — (PDF) [file pone.0295100.s002.pdf]

S1 Table. Correlations between the variables of interest (N = 1595)

	Mental Health				Burnout		
	Depression Symptoms	Suicidal Ideation	Anxiety Symptoms	Stress	Emotional Exhaustion	Cynicism	Academic Efficacy
**Mental Health**							
Depression Symptoms	1.00						
Suicidal Ideation	0.62	1.00					
Anxiety Symptoms	0.75	0.49	1.00				
Stress	0.62	0.35	0.58	1.00			
**Burnout**							
Emotional Exhaustion	0.62	0.41	0.55	0.53	1.00		
Cynicism	0.42	0.43	0.35	0.25	0.50	1.00	
Academic Efficacy	-0.46	-0.41	-0.47	-0.28	-0.46	-0.54	1.00

Note. Depression symptoms was measured with the Center for Epidemiological Studies-Depression (CES-D), suicidal ideation with two questions of the Beck Depression Inventory (BDI), anxiety symptoms with the trait section of the State-Trait Anxiety Inventory (STAI), stress with a single generic item (“Globally, how would you evaluate your current stress level on a scale from 1 ‘none’ to 10 ‘extreme’?”), and burnout with the Maslach Burnout Inventory Student-Survey (MBI-SS).
