# Supplementary material for: Mental health and burnout during medical school: Longitudinal evolution and covariates
Source: PLoS One. 2024 Apr 16;19(4):e0295100. doi: 10.1371/journal.pone.0295100 (PMC11020803; doi:10.1371/journal.pone.0295100)
Supplement: S1 File — (PDF) [file pone.0295100.s003.pdf]

S1 File. Equations for the Linear mixed model analyses

To model the longitudinal evolution of mental health and burnout during the course of the medical school, Linear Mixed Models (LMMs) were fitted to account for the clustering in the data due to the repeated measures for each medical students, considering the student specific effects as random intercepts. Consider Y_{ij}^{k} being the kth mental health and burnout outcome then the model building process can be summarized in the following steps:

1. Model (1) was fitted using $Y_{ij}^{k} = \beta_0 + \beta_1 * Year_{ij} + B * X_{ij} + u_i + \epsilon_{ij}$, where $Year_{ij}$ indicates the curriculum year of the student (i) at his jth measurement and X_{ij} contains all the other covariates the model is adjusted for. Finally, u_i is the random intercept for student (i) that follows a normal distribution and is independent from $\epsilon_{ij} \sim N(0, \Sigma_{\mathrm{i}})$. In this model the variance covariance is considered to be $\Sigma_i = \sigma I_{ni}$, where I_{ni} is an identity matrix.
2. Model (2) was fitted considering the same model as in step (1) but where the Σ_i is considered to have Autoregressive Covariance Structure of AR(1).
3. Model (3) was fitted considering the same model as in step (1) where the Σ_i is considered to have Autoregressive/Moving Average Covariance Structure of ARMA(1,1).
4. Likelihood ratio test was used to choose the best model among (1), (2) and (3), separately for each outcome.
5. Model (4) was fitted using $Y_{ij}^{k} = \beta_0 + \beta_1 * Year_{ij} + \beta_2 * year_{ij}^{2} + B * X_{ij} + u_i + \epsilon_{ij}$, with the chosen structure of Σ_i.
6. Model (5) was fitted using $Y_{ij}^{k} = \beta_0 + \beta_1 * Year_{ij} + \beta_2 * year_{ij}^{2} + \beta_3 * year_{ij}^{3} + B * X_{ij} + u_i + \epsilon_{ij}$, with the chosen structure of Σ_i.
7. Separately for each outcome, likelihood ratio test was used to compare models (4) and (5) to model (1), (2), or (3) according to the chosen structure of Σ_i and the best fitting one was presented as the final model.
